# Testosterone Replacement Therapy as a Potential Strategy to Preserve Lean Mass in Men With Persistently Low Serum Testosterone Receiving GLP-1 Receptor Agonists: A Narrative Review

**DOI:** 10.7759/cureus.105734

**Published:** 2026-03-23

**Authors:** Luis M Canal de Velasco, José Emiliano González Flores, Gabriel Kraus Fischer, Jose Luis Morales Arteaga, María J Olivares Casas, Kenia N Alvarez Nava, Frida M Morales Morales

**Affiliations:** 1 Medical Education and Simulation, Panamerican University, Mexico City, MEX; 2 Surgery, Tecnológico de Monterrey Campus Ciudad de Mexico, Mexico City, MEX; 3 Bariatric Surgery, ABC Medical Center, Mexico City, MEX; 4 Cardiology, Panamerican University, Mexico City, MEX; 5 Medical Education and Simulation, Faculty of Medicine, National Autonomous University of Mexico (UNAM), Mexico City, MEX; 6 Medical Education and Simulation, School of Medicine, Anahuac University, Mexico City, MEX

**Keywords:** body composition, fat-free mass, glp-1 receptor agonists, lean mass, low testosterone, obesity pharmacotherapy, sarcopenia, skeletal muscle, testosterone replacement therapy

## Abstract

Glucagon-like peptide-1 receptor agonists (GLP-1 RAs) have redefined the management of obesity and type 2 diabetes mellitus, producing sustained and clinically meaningful reductions in total body weight alongside significant cardiometabolic benefits. However, weight loss achieved through pharmacologic energy deficit includes reductions in both adipose tissue and fat-free mass. Although some degree of lean mass decline is physiologically expected, its clinical relevance may vary according to age, baseline muscle reserve, physical activity, nutritional intake, and endocrine status.

Testosterone is a key regulator of skeletal muscle mass and metabolic homeostasis. In men with low testosterone levels, testosterone replacement therapy (TRT) consistently increases lean body mass and favorably modifies body composition. Notably, obesity and metabolic disease are frequently associated with reduced circulating testosterone concentrations, creating a subset of patients with diminished anabolic reserve who may be particularly vulnerable to clinically meaningful muscle loss during sustained weight reduction.

This structured narrative review examines the physiological and clinical evidence linking GLP-1 RA-induced weight loss, body composition changes, and androgen signaling. While mechanistic and clinical data independently support the anabolic role of testosterone and the predictable lean mass changes accompanying pharmacologic weight loss, no randomized trials have directly evaluated the combined effects of GLP-1 RAs and TRT on body composition outcomes.

The available evidence suggests a biologically plausible interaction but does not support routine combined therapy. Testosterone replacement should be beneficial in men with low testosterone levels according to current guidelines; assessment of androgen status, including individuals with low or borderline testosterone levels frequently observed in obesity, may be clinically relevant when evaluating patients experiencing substantial weight loss with GLP-1-based therapies. Future prospective studies incorporating standardized body composition assessment and functional endpoints are required to determine whether androgen status modifies musculoskeletal responses to GLP-1-based pharmacotherapy.

## Introduction and background

Background

Glucagon-like peptide-1 receptor agonists (GLP-1 RAs) have emerged as highly effective pharmacologic therapies for obesity and type 2 diabetes mellitus, producing substantial and sustained weight loss while improving cardiometabolic outcomes. Large randomized clinical trials such as STEP 1 and SURMOUNT-1 have demonstrated mean total body weight reductions exceeding 10-20% with semaglutide and tirzepatide, respectively, positioning these medications as central components of contemporary obesity management [1,2]. Beyond glycemic control, GLP-1-based therapies have reshaped the therapeutic landscape of metabolic disease and are increasingly prescribed across broad patient populations.

Weight reduction, however, is not confined to adipose tissue. Across multiple weight-loss interventions, including dietary energy restriction and pharmacologic therapies, a proportion of the total weight loss consists of fat-free mass, including skeletal muscle [3,4]. Analyses of GLP-1-based trials indicate that measurable reductions in lean mass accompany total weight loss, although the relative contribution varies depending on baseline characteristics and assessment methodology [1,5,6]. While some degree of lean mass decline is physiologically expected during negative energy balance, excessive or disproportionate reductions in skeletal muscle may have important clinical implications in individuals with limited anabolic reserve, defined as a reduced capacity to maintain or restore skeletal muscle mass due to diminished anabolic hormone availability and/or impaired responsiveness of muscle tissue to anabolic stimuli such as testosterone [6,7]. Loss of muscle mass and strength has been associated with impaired physical performance, metabolic dysregulation, and increased morbidity within the framework of sarcopenia and sarcopenic obesity [7,8,9]. 

Testosterone plays a central role in skeletal muscle regulation, influencing protein synthesis, myocyte differentiation, and body composition [9,10,11,12]. Experimental and clinical data demonstrate that reductions in circulating testosterone are associated with diminished lean mass and unfavorable alterations in fat distribution [13]. In men with confirmed low testosterone concentrations, testosterone replacement therapy (TRT) has been shown to increase lean body mass and improve selected muscle-related outcomes [14,15,16]. Importantly, obesity and metabolic disease are strongly associated with reduced total testosterone levels, partly related to decreased sex hormone-binding globulin (SHBG), which can influence circulating free and bioavailable testosterone fractions. This relationship appears to be bidirectional and partially mediated by adiposity and inflammatory signaling [16].

In this context, the coexistence of pharmacologically induced weight reduction and diminished anabolic signaling may represent a clinically relevant scenario. A substantial proportion of men initiating GLP-1 therapy may already exhibit reduced testosterone concentrations, raising the possibility that underlying endocrine vulnerability could influence body composition responses during sustained weight loss.

Rationale

Lean mass regulation during weight reduction reflects a complex interplay among caloric deficit, dietary protein intake, mechanical loading, and endocrine milieu. Skeletal muscle homeostasis depends on the dynamic balance between protein synthesis and protein breakdown, processes that are modulated by nutritional status and androgen signaling [10,11]. Evidence from controlled dietary studies demonstrates that energy restriction alone can lead to reductions in fat-free mass, even in the presence of structured interventions [3,4]. Within this framework, men with persistently low serum testosterone levels may exhibit diminished anabolic capacity and therefore increased susceptibility to muscle loss during periods of substantial weight reduction.

Glucagon-like peptide receptor agonists are not intrinsically catabolic agents; rather, they facilitate sustained negative energy balance and weight reduction through appetite modulation and metabolic effects. Nevertheless, clinical trials consistently report that a proportion of total weight loss consists of lean tissue [1,2,5]. Separately, multiple randomized and meta-analytic studies demonstrate that restoration of physiological testosterone levels in men with documented androgen deficiency increases lean body mass and favorably modifies body composition [14,16]. 

To date, no randomized clinical trials have directly evaluated the combined effects of GLP-1 receptor agonist therapy and testosterone replacement on body composition outcomes. However, integrating the established effects of GLP-1-associated weight loss on fat-free mass with the anabolic effects of TRT in men with low testosterone concentrations raises a clinically relevant and mechanistically plausible question: whether normalization of androgen levels in selected patients may contribute to lean mass preservation during pharmacologically induced weight reduction. Given the expanding global use of GLP-1-based therapies and the recognized prevalence of low testosterone in men with obesity and metabolic disease, this intersection represents a timely and insufficiently explored domain of investigation.

Objective

The objective of this narrative review is to examine the physiological and clinical evidence regarding the potential role of testosterone replacement therapy in preserving lean mass among men with persistently low serum testosterone levels undergoing GLP-1 receptor agonist-associated weight loss. This review synthesizes current evidence on body composition changes observed during GLP-1 receptor agonist therapy, summarizes mechanistic and clinical data on the effects of testosterone and TRT on skeletal muscle and fat-free mass, and discusses the theoretical and clinical considerations relevant to lean mass preservation in this context. Additionally, current gaps in the literature are identified to inform future research aimed at clarifying safety, patient selection, and the appropriateness of potential combined therapeutic strategies.

## Review

Methods

Study Design

This study was conducted as a structured narrative review using a structured and reproducible search strategy to synthesize current evidence regarding the potential role of testosterone replacement therapy (TRT) in preserving lean mass among men with persistently low serum testosterone levels undergoing GLP-1 receptor agonist-associated weight loss. This approach was selected due to substantial heterogeneity across study populations, intervention characteristics, outcome definitions, and body composition assessment methods, which precluded a formal systematic review or quantitative meta-analysis. The review integrates mechanistic, clinical, and translational evidence to construct a physiologically coherent framework while maintaining transparency in literature identification and selection.

Literature Search Strategy

A structured literature search was performed across PubMed/MEDLINE, Embase, Scopus, Web of Science, and the Cochrane Library to identify relevant publications addressing body composition changes during GLP-1 receptor agonist therapy, the effects of testosterone and TRT on lean mass and skeletal muscle, and the intersection between metabolic weight loss and androgen physiology. The search period extended from January 1990 through December 2025 to capture both foundational mechanistic research on testosterone signaling and contemporary clinical trials evaluating GLP-1 receptor agonists and testosterone replacement therapy. The search strategy combined Medical Subject Headings (MeSH) and free-text terms, including “testosterone replacement therapy,” “TRT,” “low testosterone,” “hypogonadism,” “GLP-1 receptor agonist,” “semaglutide,” “tirzepatide,” “lean mass,” “fat-free mass,” “skeletal muscle,” “body composition,” “weight loss,” and “sarcopenia.” Boolean operators (AND/OR) were applied as appropriate. Reference lists of key studies and review articles were manually screened to ensure completeness, and citation tracking was performed to identify additional relevant publications. Only peer-reviewed articles published in English were considered eligible.

Study Selection and Eligibility Criteria

Eligible publications included randomized controlled trials, prospective and retrospective observational studies, mechanistic and translational investigations, meta-analyses, and high-quality narrative reviews that addressed either: (1) changes in body composition during GLP-1 receptor agonist therapy, (2) the effects of testosterone or TRT on lean mass, skeletal muscle physiology, or body composition, or (3) the relationship between obesity, metabolic disease, and circulating testosterone levels. Studies exclusively evaluating supraphysiologic androgen dosing, anabolic steroid misuse, or performance enhancement in eugonadal athletes were excluded. Editorials, opinion pieces, conference abstracts, and non-peer-reviewed sources were also excluded. Because TRT indications and most outcome data are derived from male endocrine reference ranges, the evidence base included in this review was predominantly limited to adult male populations. Studies involving female cohorts were considered when relevant for mechanistic insights but were not used to extrapolate clinical conclusions regarding TRT. Titles, abstracts, and full-text articles were evaluated based on relevance to the research question. Study selection was guided by conceptual relevance and methodological quality, aiming to provide a balanced and clinically meaningful synthesis of the available evidence. This approach was intended to enhance transparency rather than to replicate the methodology of a formal systematic review.

Reporting Approach

**Figure 1:**
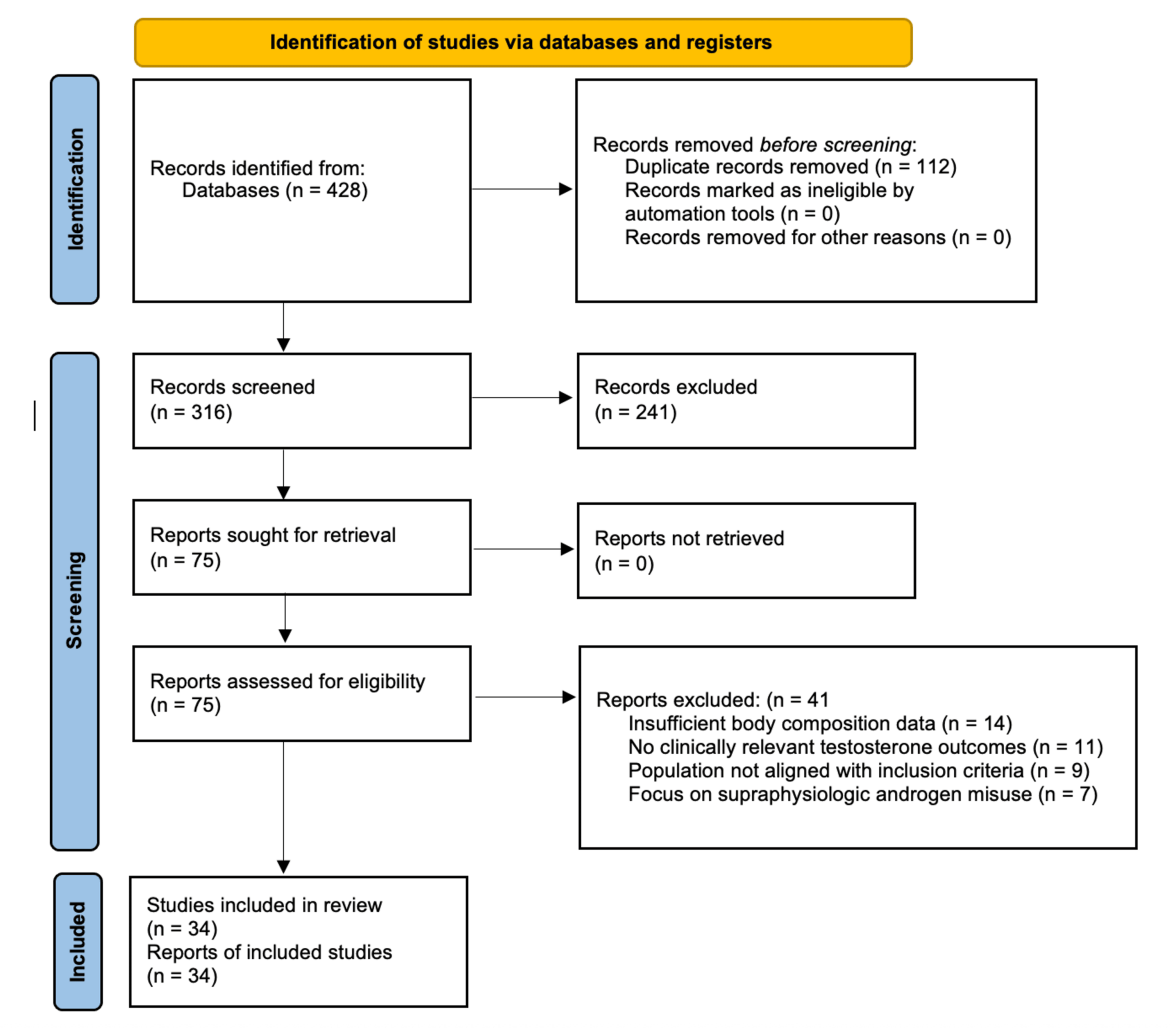
PRISMA 2020 flow diagram illustrating the study selection process for the structured narrative review. The diagram summarizes records identified through database searches, removal of duplicates, title and abstract screening, full-text eligibility assessment, and final inclusion in the qualitative synthesis. A total of 428 records were identified, 112 duplicates were removed, 316 records were screened, and 75 full-text articles were assessed for eligibility. Thirty-four studies met predefined inclusion criteria and were included in the final narrative synthesis.

To enhance methodological transparency, elements of systematic review reporting were incorporated to describe the literature identification and selection process. The literature search and selection process identified a subset of studies considered most relevant to the objectives of this review, which were included in the final qualitative synthesis. A PRISMA-inspired flow diagram (Figure 1) is provided for illustrative purposes to improve reporting clarity, without implying a formal systematic review methodology.

Quality Appraisal and Data Synthesis

Given the integrative nature of the review and inclusion of mechanistic and translational literature, formal quantitative risk-of-bias tools were not uniformly applicable. Instead, studies were qualitatively appraised according to methodological rigor, sample size, population relevance, outcome definition, and level of evidence, consistent with the Oxford Centre for Evidence-Based Medicine (2020 update) [17].

Extracted data were organized into predefined thematic domains, including: (1) body composition changes associated with GLP-1 receptor agonist therapy, (2) molecular and physiological mechanisms of testosterone signaling in skeletal muscle, (3) clinical effects of TRT on lean mass and body composition in men with low testosterone, (4) obesity-related suppression of endogenous testosterone and metabolic interactions, and (5) safety considerations relevant to combined metabolic and endocrine management.

An integrative narrative synthesis was performed to contextualize mechanistic findings alongside clinical evidence while explicitly acknowledging heterogeneity in body composition measurement methods (dual-energy X-ray absorptiometry (DEXA), BIA (bioelectrical impedance analysis), imaging-based approaches), variability in patient populations, and the absence of direct randomized trials evaluating combined GLP-1 receptor agonist and testosterone replacement therapy.

Review

Body Composition Changes During GLP-1 Receptor Agonist Therapy

Glucagon-like peptide-1 receptor agonists (GLP-1 RAs) have demonstrated robust and reproducible reductions in total body weight across large-scale randomized clinical trials in individuals with obesity and type 2 diabetes mellitus. In STEP 1, once-weekly semaglutide 2.4 mg produced a mean weight reduction of approximately 14.9% at 68 weeks compared with placebo [1], while SURMOUNT-1 reported weight reductions approaching 20% with tirzepatide at higher doses [2]. These magnitudes of weight loss approach those observed after metabolic surgery and have redefined pharmacologic management of obesity.

However, total weight loss comprises reductions in both adipose tissue and fat-free mass (FFM). DEXA sub-analyses from STEP 1 demonstrated that approximately 30-40% of total weight loss was attributable to reductions in lean mass, with the remainder reflecting fat mass loss [1]. Similar proportional changes were observed during extended treatment and maintenance phases, as reported in STEP 4 [5]. Contemporary reviews focusing on GLP-1 receptor agonists consistently report that reductions in lean mass, comprising skeletal muscle as well as other fat-free components such as body water and organ tissue, accompany pharmacologically induced weight loss, although the relative contribution varies based on baseline adiposity, age, metabolic health, and body composition assessment modality [6].

Importantly, this pattern is not unique to GLP-1-based therapies. Across dietary energy restriction and non-surgical weight loss interventions, 20-40% of total weight reduction is commonly derived from fat-free mass, even under structured nutritional protocols [3,4]. Thus, lean mass reduction during GLP-1 RAs therapy appears to reflect the physiological consequences of sustained negative energy balance rather than a direct catabolic effect of GLP-1 receptor signaling. The metabolic milieu of weight loss, characterized by reduced caloric intake, altered substrate utilization, and shifts in hormonal regulation, inevitably influences skeletal muscle protein turnover.

The clinical relevance of these changes depends not solely on absolute FFM reduction but on functional implications. As emphasized by the European Working Group on Sarcopenia in Older People (EWGSOP2), sarcopenia is defined by the combination of low muscle strength and low muscle quantity or quality, with functional impairment as the central determinant of clinical significance [7]. Therefore, interpretation of lean mass decline during GLP-1-induced weight loss must consider baseline anabolic reserve, age, comorbid disease burden, and physical activity patterns. In metabolically robust individuals with preserved endocrine function, moderate proportional reductions in FFM may represent an expected and physiologically tolerable component of weight normalization. Conversely, in populations characterized by impaired anabolic signaling, such as men with persistently low serum testosterone concentrations, the same magnitude of lean mass reduction may carry amplified functional and metabolic consequences.

Collectively, current evidence indicates that GLP-1 receptor agonist therapy induces substantial total weight loss that includes predictable reductions in fat-free mass (Table 1). While this phenomenon aligns with established principles of energy balance physiology, it raises clinically relevant considerations regarding skeletal muscle preservation in subgroups with diminished anabolic capacity. Identifying patients at heightened risk for clinically meaningful lean mass decline represents an essential step in optimizing long-term metabolic and functional outcomes during pharmacologically induced weight reduction.

**Table 1:** Body Composition Changes Reported in Major GLP-1 Receptor Agonist Trials In most trials, fat-free mass reflects a composite of skeletal muscle and other non-fat tissues (e.g., body water and organ mass), and therefore should not be interpreted as a direct surrogate of functional skeletal muscle. Abbreviations: BIA, bioelectrical impedance analysis; DEXA, dual-energy X-ray absorptiometry; FFM, fat-free mass; GLP-1 RA, glucagon-like peptide-1 receptor agonist.

Trial	Agent	Mean Total Weight Loss	Lean Mass Change	Proportion of Weight Loss Attributable to Lean Mass	Assessment Method
STEP 1 [1]	Semaglutide 2.4 mg	~14.9% at 68 weeks	Significant reduction in FFM	~30–40% of total weight loss	DEXA
STEP 4 [5]	Semaglutide (maintenance phase)	Sustained weight reduction	Continued FFM changes	Proportional to total weight loss	DEXA
SURMOUNT-1 [2]	Tirzepatide	Up to ~20%	Lean mass reduction reported	Noted as a fraction of total weight loss	DEXA (subset analyses)
Systematic Review [6]	GLP-1 RAs (various)	Variable	Consistent lean mass reductions	20–40% range across studies	DEXA/BIA

Mechanisms of Lean Mass Reduction During Pharmacologically Induced Weight Loss

Reductions in lean mass during GLP-1 receptor agonist-associated weight loss are best understood within the broader physiological framework of sustained negative energy balance. Skeletal muscle homeostasis is governed by the dynamic equilibrium between muscle protein synthesis (MPS) and muscle protein breakdown (MPB). Caloric restriction shifts this balance toward net protein loss, particularly when nutrient intake and mechanical loading are insufficient to sustain anabolic signaling [1,3]. 

At the molecular level, reduced nutrient availability attenuates activation of key anabolic pathways, including mechanistic target of rapamycin (mTOR), a central regulator of translational efficiency and muscle hypertrophy. Experimental data indicate that androgen signaling positively modulates mTOR activity and satellite cell function, thereby enhancing muscle protein accretion [10,11,12]. In contrast, reduced anabolic signaling, whether due to caloric restriction, aging, or low circulating testosterone, may impair the capacity to maintain muscle mass under conditions of metabolic stress. 

Clinical studies in energy-restricted states have demonstrated measurable reductions in fat-free mass even when structured interventions are implemented [3,4]. Importantly, testosterone exerts direct effects on skeletal muscle protein metabolism. Controlled trials show that testosterone administration increases muscle protein synthesis, enhances nitrogen retention, and promotes lean mass accrual in men with low testosterone concentrations [14,15]. Dose-response investigations further support a graded relationship between circulating testosterone levels and changes in muscle mass and strength [13,18,19]. 

Beyond direct anabolic signaling, weight loss may influence skeletal muscle through secondary mechanisms. Reduced total body mass decreases habitual mechanical loading, potentially diminishing hypertrophic stimuli. Low circulating testosterone may impair the capacity to maintain muscle mass under conditions of metabolic stress and is also associated with reduced exercise tolerance, potentially limiting the mechanical stimuli required for skeletal muscle hypertrophy and adaptation [20,21]. In parallel, obesity-associated low testosterone levels, characterized by reduced total and free testosterone levels, are common in men with obesity, metabolic syndrome, or type 2 diabetes, creating a pre-existing state of diminished anabolic reserve [18]. Observational and interventional data suggest that restoration of physiological androgen levels in such populations improves body composition and may mitigate components of metabolic dysfunction [22,23]. 

Inflammatory signaling also contributes to muscle remodeling during metabolic perturbation. Androgen replacement has been associated with reductions in pro-inflammatory cytokine production and modulation of immune cell activity [24,25,26], mechanisms that may indirectly influence skeletal muscle preservation. Additionally, testosterone has been linked to enhanced mitochondrial biogenesis and improved oxidative capacity in skeletal muscle [27], factors relevant to muscle quality beyond absolute mass. Systemic effects, including stimulation of erythropoiesis via increased erythropoietin and modulation of iron metabolism [28,29], may further affect oxygen delivery and functional performance.

Taken together, lean mass reduction during GLP-1 receptor agonist-associated weight loss is best understood as a predictable consequence of sustained negative energy balance, reduced mechanical loading, and broader metabolic adaptation rather than as a direct catabolic effect of GLP-1 receptor signaling. The clinical significance of these changes may vary according to baseline anabolic capacity. In men with persistently low serum testosterone concentrations, reduced androgen-mediated anabolic support may theoretically increase vulnerability to skeletal muscle loss during weight reduction. These parallel physiological processes provide a biologically plausible rationale for examining whether restoration of physiological testosterone levels, when clinically indicated, could influence body composition trajectories during pharmacologically induced weight loss. Table 2 summarizes theoretical mechanisms underlying these processes.

**Table 2:** Theoretical physiological mechanisms relevant to lean mass regulation during GLP-1 receptor agonist–associated weight loss and established effects of testosterone This table summarizes theoretical physiological domains involved in lean mass regulation during sustained negative energy balance and GLP-1 receptor agonist–associated weight loss, alongside established effects of testosterone on skeletal muscle biology. The mechanisms presented are derived from experimental, clinical, and translational evidence considered independently, and are intended to provide a conceptual framework rather than represent direct evidence from combined GLP-1 receptor agonist and TRT interventions. Accordingly, this table should not be interpreted as evidence of a causal interaction between GLP-1 signaling and androgen pathways or as proof of benefit from combined therapy. Abbreviations: GLP-1, glucagon-like peptide-1; MPS, muscle protein synthesis; mTOR, mechanistic target of rapamycin; RA, receptor agonist; TRT, testosterone replacement therapy.

Mechanistic Domain	Physiological Changes During Sustained Energy Deficit / GLP-1–Induced Weight Loss	Evidence Supporting Lean Mass Reduction	Potential Modulatory Role of Testosterone	Key References
Muscle Protein Turnover	Reduced caloric intake shifts the balance toward decreased net muscle protein synthesis (MPS)	Energy restriction associated with fat-free mass decline	Testosterone stimulates MPS, enhances nitrogen retention, and increases lean mass	[1,4,14,15]
mTOR and Anabolic Signaling	Nutrient scarcity attenuates mTOR activation and translational efficiency	Impaired anabolic signaling during metabolic stress	Testosterone enhances mTOR pathway activation and satellite cell function	[10,11,12,29]
Mechanical Loading	Reduction in total body mass decreases habitual mechanical stimulus to skeletal muscle	Lower loading may attenuate hypertrophic signaling	Testosterone augments muscle adaptation to resistance stimuli	[9,19]
Obesity-Associated Low Testosterone Serum Levels	Adiposity and metabolic disease suppress endogenous testosterone production	Low testosterone is linked to reduced lean mass and unfavorable body composition	Testosterone replacement therapy (TRT) restores physiological androgen levels and improves body composition	[18,22,23]
Inflammatory Signaling	Metabolic perturbation alters cytokine milieu and muscle remodeling	Chronic low-grade inflammation may impair muscle integrity	TRT reduces pro-inflammatory cytokines and modulates immune activity	[24,25,26]
Mitochondrial Function	Energy deficit alters mitochondrial turnover and oxidative metabolism	Changes in muscle quality beyond absolute mass	Testosterone associated with enhanced mitochondrial biogenesis	[27]
Body Composition Regulation	Weight loss includes predictable reductions in fat-free mass	Lean mass reduction reported in GLP-1 RA trials	TRT increases lean body mass in men with low testosterone	[1,2,13,16]
Functional and Systemic Adaptation	Changes in oxygen delivery and metabolic efficiency during weight loss	Potential impact on muscle performance	Testosterone stimulates erythropoiesis and oxygen-carrying capacity	[21]

Testosterone Physiology and Skeletal Muscle Regulation

Testosterone is a central regulator of skeletal muscle mass, composition, and functional capacity. Androgen signaling in muscle tissue occurs primarily through binding to the intracellular androgen receptor (AR), which subsequently modulates transcription of genes involved in protein synthesis, satellite cell activation, and myofibrillar hypertrophy. Experimental data demonstrate that testosterone enhances muscle protein synthesis, increases nitrogen retention, and promotes myogenic differentiation, thereby contributing to the maintenance of lean mass across the adult lifespan [10,11,12]. Randomized clinical trials and meta-analytic data consistently demonstrate that testosterone therapy increases lean body mass in men with low testosterone concentrations [15,16,19]. Additional clinical syntheses and integrative perspectives have been described in prior literature [30,31]. 

At the molecular level, testosterone influences key anabolic pathways, including activation of the mechanistic target of rapamycin (mTOR) complex and downstream translational machinery. These effects facilitate increased incorporation of amino acids into contractile proteins and support satellite cell proliferation and fusion, processes essential for muscle hypertrophy and regeneration [11,29]. In parallel, testosterone exerts anti-catabolic effects by modulating ubiquitin-proteasome activity and reducing proteolytic signaling, thereby influencing the net balance between muscle protein synthesis and breakdown [10,11,15,29].

Clinical investigations provide convergent evidence of the relationship between circulating testosterone concentrations and body composition. In dose-response studies, graded reductions in serum testosterone were associated with progressive decreases in lean body mass and increases in fat mass, independent of age [13]. Conversely, administration of testosterone in men with low endogenous levels increases lean mass and improves selected measures of muscle strength and physical function [15,19]. Meta-analytic data further support consistent increases in lean body mass among men receiving TRT, although improvements in functional outcomes may vary depending on baseline characteristics and study duration [16]. Beyond musculoskeletal endpoints, systematic reviews and clinical studies have also examined broader neurocognitive and behavioral dimensions of testosterone therapy [3,32,33,34], with additional perspectives described in prior literature [32].

Beyond absolute muscle mass, testosterone appears to influence muscle quality and metabolic function. Evidence suggests that androgen signaling contributes to mitochondrial biogenesis and oxidative capacity within skeletal muscle fibers [25,27], potentially affecting fatigue resistance and metabolic efficiency. Additionally, testosterone modulates erythropoiesis through stimulation of erythropoietin production and suppression of hepcidin, thereby enhancing oxygen-carrying capacity [21]. These systemic effects may have functional implications beyond changes in muscle size alone.

Importantly, low serum testosterone levels are common in men with obesity and metabolic syndrome, conditions frequently treated with GLP-1 receptor agonists. Observational data indicate that obesity-associated low testosterone serum levels are linked to unfavorable body composition and reduced lean mass [18]. Interventional studies suggest that restoration of physiological androgen levels in such populations improves lean mass and may favorably influence metabolic parameters [22,23]. However, testosterone therapy is not indicated for weight loss per se and must be prescribed according to established clinical guidelines in men with confirmed biochemical and clinical evidence of androgen deficiency [19,33].

**Figure 2:**
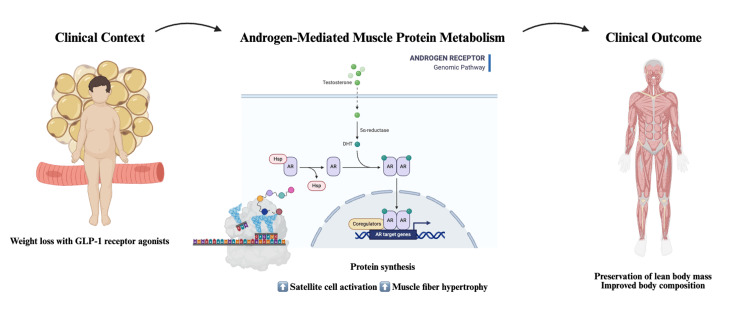
Proposed mechanistic framework linking androgen signaling and preservation of lean body mass during GLP-1 receptor agonist–induced weight loss. Glucagon-like peptide-1 (GLP-1) receptor agonists promote significant weight loss, which may involve reductions in both adipose tissue and lean body mass. In individuals with reduced androgen activity, diminished anabolic signaling may contribute to skeletal muscle loss. Testosterone and its metabolite dihydrotestosterone (DHT) activate the androgen receptor (AR), promoting transcription of target genes involved in muscle protein synthesis, satellite cell activation, and muscle fiber hypertrophy. These processes may contribute to the preservation of lean body mass and improved body composition during pharmacologically induced weight loss. The colored blocks within the DNA schematic represent androgen-responsive target genes regulated by AR-mediated transcription. Abbreviations: AR, androgen receptor; HSP, heat shock proteins; DHT, dihydrotestosterone. This figure was created by the authors using BioRender (BioRender, Toronto, Canada).

Collectively, current physiological and clinical evidence strongly supports a biologically meaningful relationship between androgen signaling and skeletal muscle regulation. Testosterone influences muscle mass through direct anabolic effects, modulation of protein turnover pathways, and systemic metabolic mechanisms. In men with persistently low serum testosterone levels, diminished anabolic signaling may represent a state of reduced muscle resilience under conditions of metabolic stress. This biological framework supports the hypothesis that androgen status may influence skeletal muscle resilience during sustained weight reduction, as illustrated in Figure 2. However, current evidence does not establish that testosterone directly modifies GLP-1 receptor signaling or that TRT specifically preserves lean mass during GLP-1 receptor agonist therapy. At present, this question remains biologically plausible but clinically unproven.

Obesity, Low Testosterone, and Anabolic Vulnerability During Weight Loss 

Obesity and metabolic disease are consistently associated with reduced circulating testosterone concentrations, a phenomenon often described as functional or obesity-related low testosterone serum levels. Large observational and longitudinal studies demonstrate that men with obesity have significantly lower total testosterone levels compared with lean counterparts, while free testosterone concentrations may be relatively preserved due to obesity-associated reductions in sex hormone-binding globulin (SHBG), with hormonal suppression correlating with visceral adiposity and metabolic dysfunction [18]. Excess adiposity contributes to dysregulation of the hypothalamic-pituitary-gonadal axis through increased aromatization of androgens to estradiol within adipose tissue, which may suppress hypothalamic-pituitary gonadotropin signaling, along with inflammatory signaling and alterations in sex hormone-binding globulin dynamics.

This endocrine environment has important implications for body composition. Reduced androgen signaling is associated with decreased lean mass and increased fat mass, independent of age [13]. Meta-analytic data further confirm that restoration of physiological testosterone concentrations in men with documented deficiency increases lean body mass and improves body composition parameters, although exogenous testosterone administration may also increase circulating estradiol levels through aromatization [16]. Observational registry studies suggest that long-term testosterone therapy in hypogonadal men may be associated with favorable changes in weight and metabolic markers [22,23]. 

Glucagon-like peptide receptor agonists are increasingly prescribed in precisely this population - men with obesity, insulin resistance, and cardiometabolic disease - among whom reductions in testosterone are common. While weight loss itself may modestly improve endogenous testosterone levels in some individuals, normalization is not universal, and a subset of men may enter a sustained period of negative energy balance with persistently reduced androgen signaling [18]. 

From a physiological perspective, sustained caloric deficit in the presence of diminished androgen-mediated anabolic signaling may predispose certain individuals to greater lean mass decline relative to those with preserved anabolic reserve. Testosterone deficiency is associated with reduced anabolic responsiveness, impaired muscle protein synthesis capacity, and unfavorable body composition patterns. Importantly, this does not imply that GLP-1 receptor agonists are intrinsically catabolic or that they directly suppress androgen pathways. Rather, the endocrine context in which weight loss occurs may influence the extent to which skeletal muscle is preserved.

Clinically, this intersection requires careful consideration. Recent cardiovascular safety data further support the importance of appropriate patient selection and monitoring [33,34,35]. Therefore, while the coexistence of obesity-related low testosterone levels and GLP-1-induced weight loss provides a biologically plausible framework for anabolic vulnerability, prospective studies are required to determine whether targeted correction of androgen deficiency modifies lean mass outcomes during pharmacologically induced weight reduction.

Clinical Implications, Evidence Gaps, and Future Directions

The intersection between GLP-1 receptor agonists-induced weight loss and obesity-associated reductions in testosterone concentrations represents a clinically relevant but insufficiently explored domain. While GLP-1-based therapies produce substantial improvements in cardiometabolic outcomes, the accompanying reductions in lean mass observed during sustained weight loss warrant individualized consideration, particularly in men with documented androgen deficiency.

At present, no randomized clinical trials have directly evaluated the combined effects of GLP-1 receptor agonists and TRT on body composition outcomes. Existing evidence derives from separate bodies of literature: (1) trials demonstrating that GLP-1 receptor agonists induce significant total weight loss with predictable reductions in fat-free mass [1,2,36,37,38], and (2) randomized and meta-analytic data indicating that TRT increases lean body mass in men with low testosterone levels [14,16]. Integrating these findings provides a mechanistic rationale for hypothesis generation but does not constitute evidence for routine combined therapy.

Clinical decision-making must therefore remain anchored in established endocrine principles. Moreover, although recent cardiovascular safety data have provided reassurance in appropriately selected populations [33], careful risk stratification and longitudinal monitoring remain essential components of TRT management.

Future research should prioritize prospective, controlled investigations evaluating body composition trajectories in men with confirmed low testosterone initiating GLP-1 receptor agonist therapy. Such studies should incorporate standardized assessment of body composition together with measures of muscle quality and function, including DEXA-derived fat-free mass, handgrip strength, chair stand performance, gait speed, and other clinically meaningful performance endpoints. Stratified analyses based on baseline testosterone concentrations may help clarify whether androgen status modifies the proportion of weight loss attributable to lean tissue. Additionally, mechanistic studies should further clarify how androgen status may modify body composition responses during GLP-1 receptor agonist-associated weight loss, without presuming a direct interaction between GLP-1 signaling and androgen pathways.

**Figure 3:**
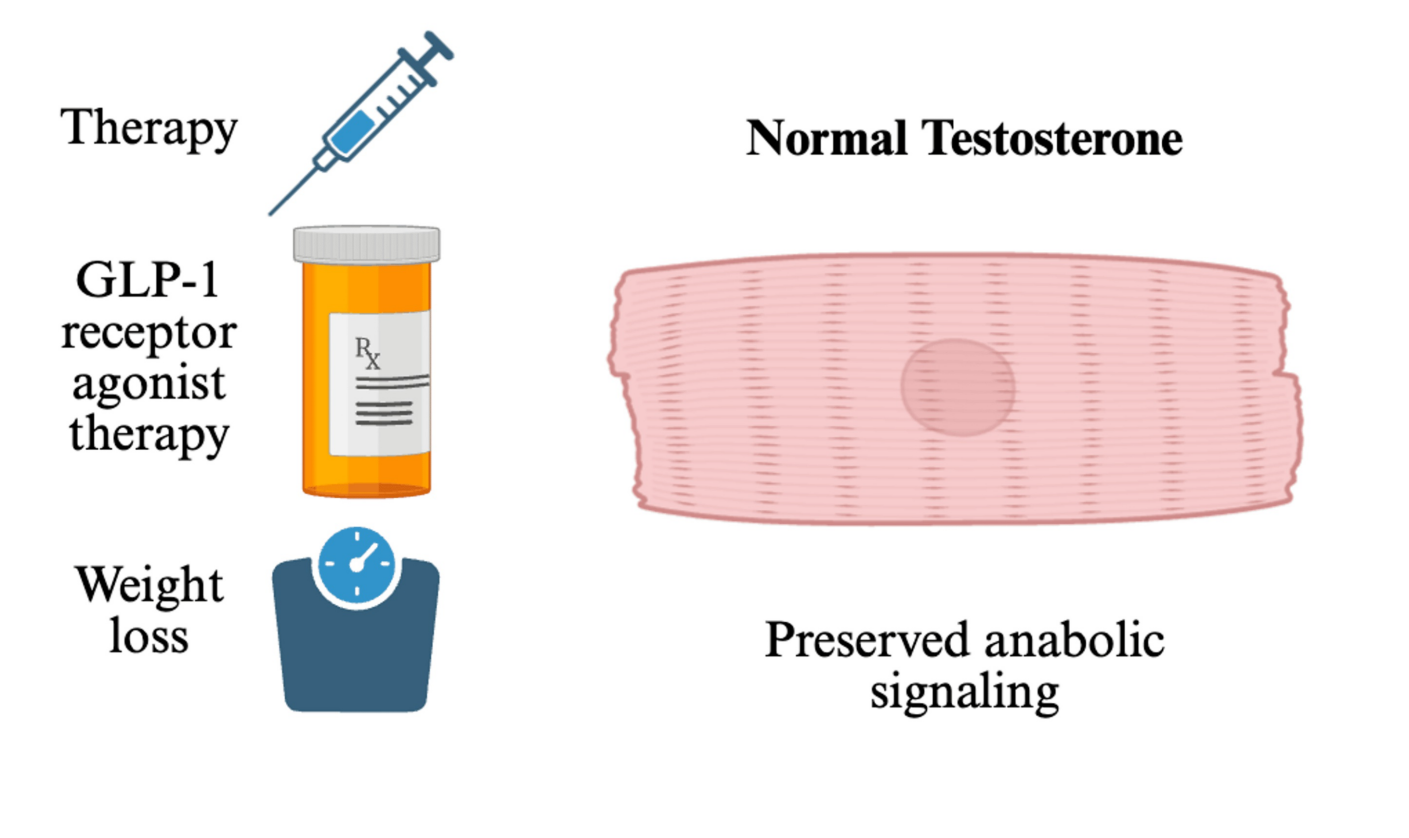
Conceptual clinical model linking GLP-1 receptor agonist therapy, testosterone status, and lean mass outcomes. Glucagon-like peptide-1 (GLP-1) receptor agonist therapy induces significant weight loss that may involve reductions in both adipose tissue and lean body mass. In individuals with preserved testosterone levels, anabolic signaling may remain relatively stable, supporting maintenance of skeletal muscle mass. In contrast, individuals with low testosterone levels may exhibit reduced anabolic reserve, potentially increasing vulnerability to lean mass decline during sustained pharmacologic weight reduction. This figure was created by the authors using BioRender (BioRender, Toronto, Canada).

In summary, while GLP-1 receptor agonists have transformed obesity management, recognition of endocrine heterogeneity among treated individuals underscores the importance of individualized assessment. In men with pre-existing or persistently low serum testosterone levels before GLP-1 therapy, restoration of physiological androgen concentrations, when clinically indicated, may help address pre-existing anabolic vulnerability during sustained weight loss. However, whether this translates into meaningful preservation of lean mass specifically during GLP-1 receptor agonist therapy remains unknown. However, this hypothesis requires validation through rigorously designed clinical trials before broader clinical adoption can be considered. The proposed clinical and research framework summarizing this conceptual intersection is illustrated in Figure 3.

Discussion

Importantly, current evidence does not support a direct causal interaction between GLP-1 receptor signaling and androgen pathways in humans. Rather, these systems appear to influence body composition through distinct but potentially convergent physiological mechanisms. Changes in testosterone observed during weight loss are more likely related to reductions in adiposity, improvements in insulin resistance, and broader metabolic adaptation than to a direct pharmacologic effect of GLP-1 receptor agonists on the hypothalamic-pituitary-gonadal axis. GLP-1 receptor agonists have reframed obesity care by delivering weight loss magnitudes that previously required bariatric procedures or highly structured lifestyle programs, but their success has re-exposed an old tension in weight management: improving cardiometabolic risk while protecting functional tissue. In STEP 1 and SURMOUNT-1, semaglutide and tirzepatide produced large, durable reductions in body weight, setting a new efficacy benchmark for pharmacotherapy [1,2]. The discussion is no longer whether weight loss is achievable, but how to interpret the accompanying remodeling of lean tissue, especially when pharmacologic weight loss is deployed at scale in populations already vulnerable to sarcopenia or low anabolic reserve [7,8]. 

A key debate is whether lean mass decline in GLP-1-treated patients should be framed as an adverse event or as an expected physiological corollary of negative energy balance. The dietary literature has long shown that fat-free mass decreases accompany intentional weight loss, and that the proportion is modifiable by protein intake and activity patterns [3,4]. This context matters because it suggests GLP-1 therapy may not be uniquely “catabolic,” but rather an efficient inducer of sustained energy deficit. However, extrapolating from classic diet trials to GLP-1 pharmacotherapy is imperfect: appetite suppression changes habitual intake patterns, and the degree to which patients spontaneously maintain protein targets and resistance loading during GLP-1 therapy is variable in real-world practice. Newer observational work highlights that physical activity and dietary intake are strongly associated with fat-free mass preservation during weight loss, reinforcing that “lean loss” is not destiny but a modifiable outcome [38]. Against that backdrop, contemporary reviews specifically examining GLP-1 agents and body composition appropriately emphasize heterogeneity across trials, measurement approaches (DEXA vs BIA), and baseline phenotypes [6,28]. The practical implication is that a given percentage of lean mass decline may be clinically inconsequential in some patients but functionally meaningful in others, particularly older adults and those with sarcopenic obesity, where the boundary between expected adaptation and clinically meaningful sarcopenia depends more on muscle strength and physical performance than on lean mass alone, consistent with EWGSOP2 definitions [7,8]. 

A critical conceptual distinction in this field is that lean mass and skeletal muscle are not interchangeable terms. Lean mass, particularly when derived from DEXA-based body composition analysis, includes all non-fat components of the body, such as skeletal muscle, body water, connective tissue, and organ mass. As a result, reductions in lean mass during pharmacologically induced weight loss do not necessarily indicate proportional loss of functional skeletal muscle tissue. This distinction is clinically important because preservation of mobility, strength, and metabolic resilience depends more specifically on skeletal muscle integrity than on lean mass as a composite compartment. Relatedly, contemporary sarcopenia frameworks emphasize that muscle health cannot be fully captured by quantity alone. The concept of muscle quality incorporates strength, physical performance, contractile efficiency, and metabolic function, and may decline even when absolute muscle mass is relatively preserved. In this context, reliance on body composition metrics alone may overestimate or underestimate clinical risk. Consistent with EWGSOP2, sarcopenia should be understood as a progressive skeletal muscle disorder defined primarily by low muscle strength, with reduced muscle quantity or quality used to confirm the diagnosis, and poor physical performance indicating severe disease. Accordingly, the clinical significance of lean tissue changes observed during GLP-1 receptor agonist-associated weight loss should be interpreted through a functional lens, particularly in older adults and in patients with low anabolic reserve, rather than through DEXA-derived lean mass values alone [31].

Within this debate, testosterone status becomes relevant less as a “countermeasure” to GLP-1 therapy and more as a modifier of the host response to weight loss. Testosterone’s anabolic role in skeletal muscle is mechanistically well supported, spanning androgen receptor-mediated transcription, protein synthesis signaling, and effects on muscle regeneration and quality [10,11,12,29]. Clinically, dose-response data show that reductions in gonadal steroids translate into graded losses of lean mass and increases in fat mass, providing a biologic foundation for the concept of reduced anabolic reserve in men with persistently low testosterone [13]. Trials and physiologic studies further indicate that testosterone administration can increase lean mass and improve selected functional metrics in appropriately selected men [14,15]. Yet the literature also cautions against overinterpreting lean mass gains as synonymous with functional restoration, a distinction particularly salient when evaluating outcomes through DEXA alone rather than paired strength and performance measures [7,28]. This is the crux: the argument for considering androgen status during pharmacologic weight loss is strongest when framed around function and vulnerability, who is at risk of clinically meaningful decline, rather than the pursuit of “more lean mass” as an endpoint.

Another clinically important tension is diagnostic and operational: “low testosterone patients” is a practical label, but variability in diagnostic criteria across guidelines and specialty practices can complicate patient selection and reproducibility across studies [36,37]. This matters because any future trials at the GLP-1/TRT intersection will be judged on whether low testosterone serum levels are rigorously defined (symptoms plus biochemical confirmation), whether measurements are repeated and standardized, and whether confounding reversible contributors (e.g., acute illness, medication effects, sleep disturbance, severe caloric restriction, or increased aromatization associated with excess adiposity) are addressed. Professional society guidelines (e.g., Endocrine Society and American Urological Association) emphasize testosterone replacement therapy only for men with confirmed biochemical and clinical testosterone deficiency, reinforcing that testosterone should not be positioned as a general adjunct to weight loss or body recomposition in eugonadal men [19,35]. In other words, the plausible mechanistic rationale does not relax the diagnostic threshold.

Safety considerations add another layer to the debate, especially when TRT is considered in metabolically complex patients who are also candidates for GLP-1 therapy. Testosterone’s predictable erythropoietic effects may be beneficial for oxygen delivery but can also produce erythrocytosis and therefore require monitoring strategies that are explicit in any combined-therapy research design [21]. Broader adverse event profiles have been synthesized in meta-analyses, supporting the need for careful surveillance and individualized risk-benefit assessment rather than categorical assumptions of safety or harm [34]. More recently, cardiovascular safety data from large-scale randomized evidence provide reassurance in appropriately selected populations, but these results should be interpreted as supporting careful clinical use, not as justification to expand indications [33]. Importantly, muscle-preservation narratives that ignore cardiovascular, hematologic, and neuropsychiatric dimensions risk appearing one-sided. Integrating evidence on broader TRT outcomes, including neurocognitive and psychiatric considerations, supports a more comprehensive clinical frame for decision-making in adults who may have comorbid depression, sleep disorders, or other overlapping symptoms [32].

A further point of comparison is whether TRT should be conceptualized as competing with, or complementing, first-line lean mass preservation strategies. The strongest evidence base for mitigating fat-free mass loss during weight loss continues to favor nutrition and exercise prescriptions, particularly adequate protein intake and resistance training, rather than pharmacologic add-ons [3,4,38]. TRT, by contrast, is a targeted therapy for a specific endocrine disorder. Therefore, the most defensible clinical stance is not “TRT to prevent GLP-1 lean loss,” but: optimize lifestyle-based muscle preservation universally; evaluate testosterone status when clinically indicated; and treat confirmed low testosterone serum levels according to guidelines, while acknowledging that whether TRT modifies GLP-1-associated lean mass trajectories remains unproven [6,19]. This framing also aligns with sarcopenia consensus statements that prioritize function and muscle strength as core targets [7].

Taken together, current evidence supports three balanced conclusions. First, lean mass reduction during GLP-1-induced weight loss is common and should be interpreted through the lens of baseline vulnerability, measurement method, and functional outcomes rather than mass alone [6,28]. Second, testosterone is a credible modifier of anabolic capacity, and TRT increases lean mass in men with confirmed low testosterone serum levels, but functional translation and safety monitoring remain central [13,14,33]. Third, the clinical hypothesis that TRT could preserve lean mass during GLP-1 therapy in men with persistently low testosterone is mechanistically plausible yet empirically untested; it should be treated as investigational until prospective, stratified studies incorporate standardized low testosterone serum levels definitions, rigorous body composition methods, and strength/performance endpoints [7,3].

## Conclusions

Glucagon-like peptide receptor agonists have transformed the management of obesity by producing substantial and sustained weight loss. However, reductions in total body mass frequently include measurable decreases in lean tissue, a phenomenon that may carry important clinical implications depending on baseline muscle reserve and endocrine status. Emerging physiological and clinical evidence suggests that testosterone plays a central role in skeletal muscle anabolism through pathways involving muscle protein synthesis, satellite cell activation, and androgen receptor-mediated transcriptional signaling. In individuals with reduced testosterone concentrations, diminished anabolic reserve may increase vulnerability to clinically meaningful lean mass loss during pharmacologically induced weight reduction.

Although current data support a plausible mechanistic interaction between GLP-1-mediated weight loss and androgen signaling, direct clinical evidence evaluating combined metabolic and endocrine strategies remains limited. Future prospective studies should examine the interaction between GLP-1 receptor agonist therapy, testosterone status, and longitudinal body composition outcomes. Such studies should incorporate systematic assessment of total, bioavailable, and free testosterone levels, as well as estradiol, during pharmacologically induced weight loss in order to clarify whether normalization of the endocrine milieu mitigates lean mass decline. Clarifying these relationships may help refine risk stratification, guide endocrine evaluation in selected patients, and inform integrated approaches aimed at optimizing weight loss while preserving skeletal muscle mass.
